# Supplementary material for: Proposal of Quick Diagnostic Criteria for Disseminated Intravascular Coagulation
Source: J Clin Med. 2022 Feb 16;11(4):1028. doi: 10.3390/jcm11041028 (PMC8878374; doi:10.3390/jcm11041028)
Supplement: Supplementary file 1 [file jcm-11-01028-s001.zip › jcm-1590723-supplementary.pdf]

Supplementary Table S1. Modified diagnostic criteria for DIC established by Japanese Ministry of Health, Labor and Welfare

Global coagulation tests	Platelet counts ($x10^3$/μl)	>80 but ≦ 120; 1 point, (within 24 hours ≧30% of reduction; 2 point) ＞50 but ≦ 80; 2 points
	FDP (μg/ml)	≧10 but < 20; 1 point, ≧20 but < 40; 2 points, ≧40; 3 points
	PT (PT ratio)	≧1.25 but <1.67; 1 point, ≧1.67; 2 points
	Bleeding symptom	Positive; 1 point
	Organ failure	Positive; 1 point
	Underlying disease of DIC	Positive; 1point
Diagnosis of DIC		≥ 7 points

DIC, disseminated intravascular coagulation; FDP, fibrinogen and fibrin degradation products; PT, prothrombin time
